# Expanded olfactory system in ray-finned fishes capable of terrestrial exploration

**DOI:** 10.1186/s12915-023-01661-8

**Published:** 2023-07-31

**Authors:** Demian Burguera, Francesco Dionigi, Kristina Kverková, Sylke Winkler, Thomas Brown, Martin Pippel, Yicheng Zhang, Maxwell Shafer, Annika L. A. Nichols, Eugene Myers, Pavel Němec, Zuzana Musilova

**Affiliations:** 1 Department of Zoology, Faculty of Science, Charles University, Prague, Czech Republic; 2 Max Planck Institute of Molecular Cell Biology and Genetics, Dresden, Germany; 3 Biozentrum, University of Basel, Basel, Switzerland

**Keywords:** Sensory evolution, Amphibious fishes, Olfactory receptors, Evolutionary transition

## Abstract

**Background:**

Smell abilities differ greatly among vertebrate species due to distinct sensory needs, with exceptional variability reported in the number of olfactory genes and the size of the odour-processing regions of the brain. However, key environmental factors shaping genomic and phenotypic changes linked to the olfactory system remain difficult to identify at macroevolutionary scales. Here, we investigate the association between diverse ecological traits and the number of olfactory chemoreceptors in approximately two hundred ray-finned fishes.

**Results:**

We found independent expansions producing large gene repertoires in several lineages of nocturnal amphibious fishes, generally able to perform active terrestrial exploration. We reinforced this finding with on-purpose genomic and transcriptomic analysis of *Channallabes apus*, a catfish species from a clade with chemosensory-based aerial orientation. Furthermore, we also detected an augmented information-processing capacity in the olfactory bulb of nocturnal amphibious fishes by estimating the number of cells contained in this brain region in twenty-four actinopterygian species.

**Conclusions:**

Overall, we report a convergent genomic and phenotypic magnification of the olfactory system in nocturnal amphibious fishes. This finding suggests the possibility of an analogous evolutionary event in fish-like tetrapod ancestors during the first steps of the water-to-land transition, favouring terrestrial adaptation through enhanced aerial orientation.

**Supplementary Information:**

The online version contains supplementary material available at 10.1186/s12915-023-01661-8.

## Background

The olfactory organs of vertebrates contain numerous sensory neurons dedicated to the perception of different chemical signals. Each of those cells is specialized in the detection of particular odorant molecules through transcriptional activation of only one among all olfactory receptor genes [1, 2]. Importantly, neurons expressing the same receptor project their axons to a specific region within the olfactory bulb (OB), establishing synapses with the mitral cells located in the same glomerulus [3]. This neural organization allows the decoding of the chemosensory input into topological information in the brain. Interestingly, great variation in the relative size of the OB is observed among vertebrate species, which has been often interpreted as a proxy for smell abilities [4–6]. However, whether the information-processing capacity of the OB is connected to the genetic variability of the olfactory receptor gene repertoire has remained largely unexplored due to the absence of high-quality genome assemblies for many relevant phylogenetic clades until recently.

At the molecular level, a few types of chemoreceptor genes acquired roles in the perception of smell during the evolution of early vertebrates [7]. As a result of these co-options, olfaction in this lineage is mainly mediated by four separate receptor families: odorant receptors (ORs), trace-amine associated receptors (TAARs), vomeronasal type 1 receptors (V1Rs) and vomeronasal type 2 receptors (V2Rs). Earlier comparative studies on these olfactory receptor genes (for simplicity, hereinafter jointly referred to as OLF genes) reported striking differences in the number of duplicates among species [8–10]. In particular, studied fishes showed a smaller number of receptors compared to many terrestrial vertebrates, supporting the idea that organisms living in aerial and water environments present distinct chemoreception needs [11]. Nevertheless, high repertoire variability has been recently detected among fishes [12–14], although selective pressures explaining these differences based on both genomic and ecological data have been rarely proposed [15].

Here, we investigated the relationship between the number of OLF genes with potentially linked phenotypic and ecological traits in ray-finned fishes. After identifying OLF genes in around two hundred high-quality genome assemblies, we characterized the evolution of their genomic organization in those lineages with greatly expanded loci. In addition, we studied the transcriptomic abundance in the olfactory organ of the different receptor families and gene clusters in a subset of organisms with repertoires varied in size. Furthermore, to investigate the physiological impact of the genomic accumulation of chemoreceptors, we also examined the association between the quantity of OLF genes and the proportion of OB cells in the brain of twenty-four species. Last, we analysed several ecological factors influencing the size of the OLF gene repertoire, finding the strongest effect in fishes able to perform terrestrial exploration to move between water bodies at night.

## Results

### Independent olfactory gene expansions in ray-finned fishes

**Fig. 1:**
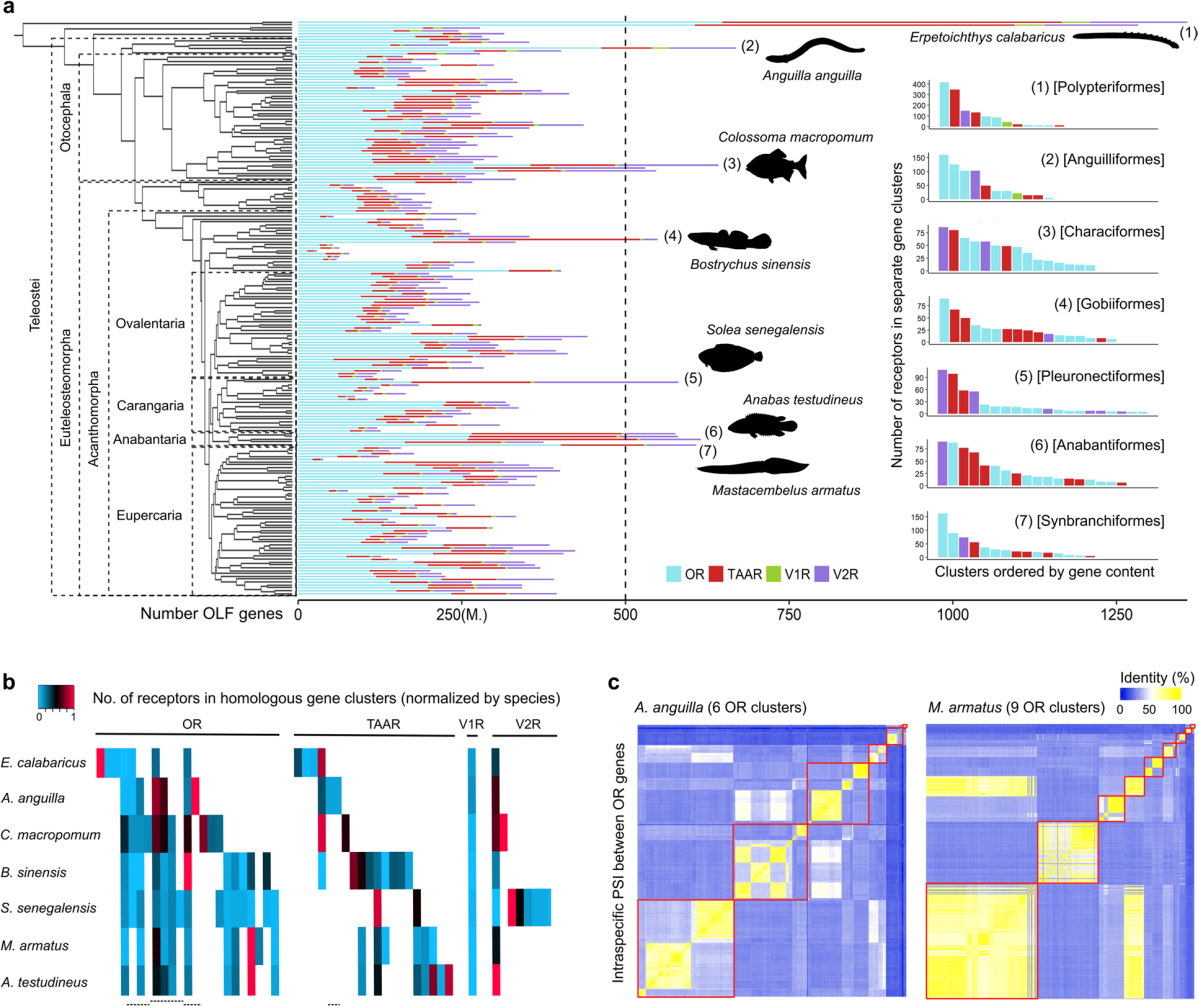
Independent OLF gene expansions in ray-finned fishes. **a** Total number of OLF genes in 201 ray-finned fish species, coloured by gene family (Additional file 2: Table S1). Median value is indicated (M.). Right, number of OR, TAAR, V1R and V2R receptors found in different genomic clusters for those species with the higher number of genes within each of the taxonomic orders presenting more than 500 OLF genes (Additional file 2: Table S2). Loci containing less than five receptor genes are excluded for visualization purposes. **b** Number of receptor genes in each genomic cluster, normalized by species. Homologous gene clusters (cells) are displayed in columns. White cells represent the absence of homologous gene clusters. Dashed lines indicate clusters of traceable homologous origin physically separated by genomic rearrangements. Loci containing less than five receptor genes in all species are excluded from the plot. **c** Heatmaps showing intraspecific pairwise sequence identity (PSI) between individual OR receptors plotted following their genomic order in the European eel (*A. anguilla*) and zig-zag eel (*M. armatus*). Gene clusters are delimited by red squares. While higher sequence identity is generally restricted to receptors from the same clusters, the zig-zag eel presents a lineage-specific cluster containing OR genes very similar to the ones located in the larger locus. Loci containing less than five OR genes are excluded

We investigated the evolutionary dynamics of the OLF genes in a large and diverse set of ray-finned fish species. We identified OR, TAAR, V1R and V2R genes in high-quality genome assemblies of more than two hundred species from approximately fifty taxonomic orders (Fig. 1a). While half of the species presented less than 250 OLF genes in total, we found a striking variability ranging from less than 30 genes in the common seadragon (*Phyllopteryx taeniolatus*) to more than 1300 in the ropefish (*Erpetoichthys calabaricus*). At the lower end, we identified eleven species from six separate clades with notably reduced repertoires (< 100 OLF genes), although most of these fishes constitute solitary cases within their taxonomic orders (Additional file 1: Fig. S1a). For example, we found dramatic variation within flatfishes (Pleuronectiformes), with almost a 7- fold difference in gene numbers between two extreme species. On the contrary, all studied pipefishes and seahorses (Syngnathiformes) present consistently miniaturized OLF families, showing evolutionary stability around diminished olfactory abilities in this singular lineage.

At the top end, we observed large repertoires of more than twice the median value (> 500 OLF genes) in species from seven distinct orders. In particular, lineages presenting such expansions include bichirs, true eels, characins, sleeper gobies, true soles, labyrinth fishes and spiny eels (Fig. 1a). Remarkably, the total number of receptor genes in the bichirs is comparable to many olfactory-oriented mammals [16]. Moreover, none of these species experienced lineage-specific whole genome duplications, suggesting that olfactory gene dynamics are fast and probably associated with macroevolutionary ecological drifts. Interestingly, despite few methodological differences (Additional file 1: Fig. S1b-e, see Methods), a recent parallel study on ray-finned fishes involving many of the same species (~ 55%) reported similar results in terms of OLF gene family expansions and contractions across this clade [14], supporting the robustness of the analysis.

As OLF genes are organized in gene clusters, we next investigated the genomic architecture of the detected expansions. In species with extensive repertoires, we observed an uneven distribution in the number of genes per cluster, with a few loci accumulating a high amount of receptors (Fig. 1a, right). These local inflations affected OR, TAAR, V1R and V2R unequally in each lineage, producing specific amplification patterns involving distinct families (Additional file 1: Fig. S2a-b) and gene clusters (Fig. 1b). We also found the number of gene clusters variable among distantly related clades due to gains, losses and/or genomic rearrangements (Fig. 1b). Because expansions are generated by tandem duplications, groups of receptors from the same gene clusters usually present the highest sequence similarities within species (Fig. 1c). As an exception, lineage-specific clusters of very recent evolutionary origin also contain receptors remarkably similar to genes from remote clusters, revealing the colonization of new genomic loci (Fig. 1c, right). However, identical receptors are generally scarce in the studied species, indicating the prevalence of coding sequence divergence after duplication events (Additional file 1: Fig. S2c).

### Genome dynamics modulate the global transcriptomic abundance of receptor genes in the olfactory organ

**Fig. 2:**
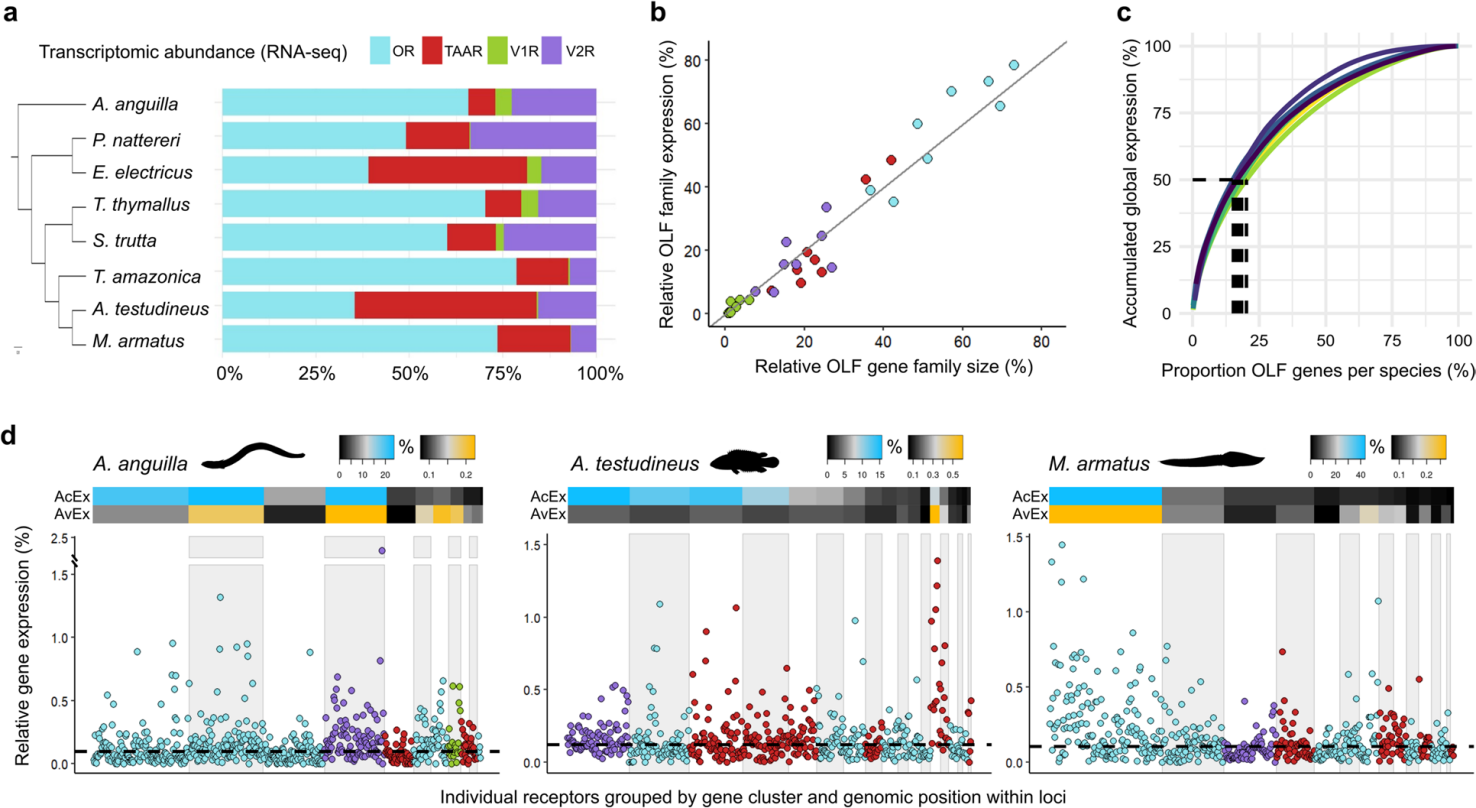
OLF genes transcriptomic abundances affected by gene cluster dynamics. **a** Relative transcriptomic abundance of each OLF family in the olfactory organ of eight sequenced species. Species in the plot are, from top to bottom: *Anguilla Anguilla*: 669 genes, *Pygocentrus nattereri*: 530 genes, *Electrophorus electricus*: 332 genes, *Thymallus thymallus*: 85 genes, *Salmo trutta*: 183 genes, *Thalassophryne amazonica*, 141 genes, *Anabas testudineus*: 615 genes and *Mastacembelus armatus*: 608 genes. Expression values correspond to the mean of two biological replicates in each species. **b** Relative repertoire size and its transcriptomic abundance is shown for each family from the eight described species. Gray line indicates a hypothetical perfect correspondence. **c** Proportion of individual receptors accumulating half of the total OLF gene expression is indicated with dashed lines for each species. Values range from around 15% in the brown trout (*S. trutta*) to approximately 20% in the climbing perch (*A. testudineus*). **d** Relative gene expression levels of individual receptors in three selected species displayed in genomic order within clusters. Gene families are coloured as in panels a and b. Median value is indicated by a dashed line. OLF gene clusters are highlighted in white and light grey alternatively. On top, heatmaps showing the percentage of global accumulated OLF expression (AcEx) and average per gene expression (AvEx) in each gene cluster

The four OLF gene families are expressed in a mutually exclusive manner in the sensory neurons of the olfactory epithelium in ray-finned fishes [17, 18]. To study how gene number evolution affects the transcriptional balance between receptors in the olfactory organ, we sequenced and analysed the transcriptomes of eight species with differently sized repertoires. We found substantial variation in the transcriptomic abundance of OR, TAAR, V1R and V2R families among lineages, roughly matching their genomic proportions (Fig. 2a, b). Moreover, we observed a minority of individual receptors (15–20%) accumulating half of the overall OLF gene expression within species, revealing the heterogeneity in the transcriptional activity of singular genes (Fig. 2c).

To investigate whether expanded gene clusters are represented at the transcriptomic level, we focused on the three species with greater OLF repertoires (> 600 genes) in more detail (Fig. 2d). We found the largest gene cluster in the zig-zag eel (*Mastacembelus armatus*) as the most abundant in terms of both accumulated and average gene expression. Similarly, the other two fishes concentrate higher fractions of the accumulated gene expression in their bigger clusters. Nevertheless, these latter species showed notably elevated transcriptional rates per gene in clusters other than the largest, with the remarkable case of a small but extremely expressed TAAR cluster in the climbing perch (*Anabas testudineus*). Thus, transcriptomic abundances at the family and cluster level globally reflect gene expansions, despite regulatory mechanisms able to enhance the expression of individual receptors in a locus-specific manner.

### Olfactory evolution linked to ecological factors and OB processing capacity

**Fig. 3:**
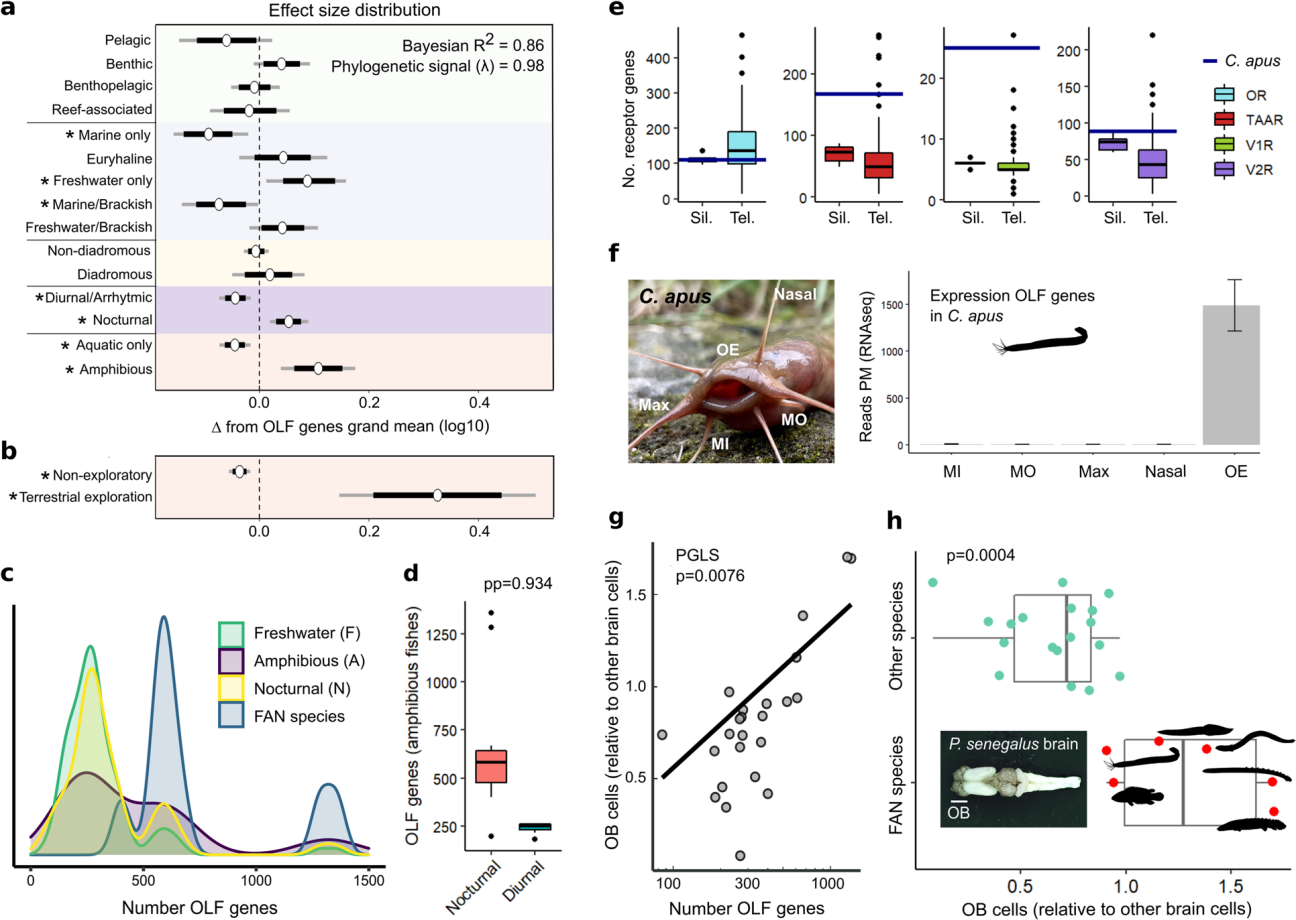
Ecological factors influencing the evolution of the OLF gene repertoire in ray-finned fishes. **a**, **b** Effect size distribution of several ecological traits on the number of OLF genes, showing highest density intervals (HDI) of 80% (black) and 95% (grey). Colours delimit related factors containing exclusive sets of species. Statistical significance (95% HDI does not include 0) is marked with an asterisk (Additional file 2: Table S4-S6). **c** Density plot with the number of OLF genes in non-exclusive ecological groups (freshwater, *n* = 125; amphibious, *n* = 19; nocturnal, *n* = 62), plus a subset of fishes presenting the three aforementioned traits (FAN, *n* = 9). **d** Differences in the OLF repertoire size between nocturnal (*n* = 11) and diurnal (*n* = 8) amphibious fishes. Posterior probability (pp) of nocturnal being higher than diurnal is indicated. **e** Number of genes for each receptor family identified in our genome assembly of *Channallabes apus* (*C. apus*), compared to other Siluriformes (Sil.; *n* = 9) and teleosts (Tel.; *n* = 195). **f** Left, picture of *C. apus* showing the position of the olfactory epithelium (OE) and the four types of barbels: nasal, maxillary (Max), mandibular outer (MO) and mandibular inner (MI). Right, number of RNA-seq reads per million (PM) from barbels and OE mapped to annotated OLF genes in *C. apus* (SD shown). **g** PGLS model showing a significant correlation between the number of OLF genes and the covariance in the number of OB and other brain cells (Additional file 2: Table S7) in twenty-four species (*x*-axis is log-transformed). **h** Significantly higher proportion of OB cells in FAN species (*n* = 6) compared to the other fish species (*n* = 18) after phylogenetic and brain size correction

To identify environmental causes influencing the number of OLF genes in ray-finned fishes, we estimated the effect of distinct ecological, life-history and behavioural traits with a potential impact on smell evolution (see the “Methods” section). We found a significant deviation towards larger repertoires in three of the studied factors: freshwater habitats, nocturnal activity and amphibious behaviour (Fig. 3a and Additional file 1: Fig. S3). Furthermore, we observed that fishes presenting the exact combination of these three traits include many of the species with greater repertoires in our data set (Fig. 3c). Interestingly, most species from this subset are also reported to perform active terrestrial exploration to find new water bodies or prey [19, 20]. Indeed, testing the influence of this type of land exploration capacity on the number of receptors as a single trait revealed the largest effect detected with our model (Fig. 3b). In contrast, diurnal amphibious fishes such as mudskippers, bullheads or some toothcarps, rarely found more than a few meters from water [21], do not present enlarged repertoires (Fig. 3d). In fact, some of these diurnal species are described to use vision to return to the aquatic environment [22, 23]. Thus, we suggest that nocturnal amphibious lineages in particular might benefit from an expansion of their OLF gene repertoire, especially during terrestrial exploratory excursions involving relatively large distances.

Terrestrial orientation through aerial chemoreception in fishes has been recently reported for the first time in a clariid species [24]. This group of catfishes, originally absent in our data set, is known to emerge from water nocturnally to move between ponds or streams. In order to investigate whether this lineage also presents an expanded OLF repertoire, we sequenced the genome of the eel catfish (*Channallabes apus*), a tropical clariid able to hunt for terrestrial prey [25, 26]. Using our high-quality sequence assembly (N50: 43 Mb, 102 scaffolds, BUSCO: 97%) at a near-chromosome level (see Methods), we identified ~ 400 OLF genes in this species. Interestingly, while the size of the OR and V2R repertoires are in line with those of other Siluriformes, we observed clear expansions of the TAAR and V1R gene families compared to both catfish and teleost species (Fig. 3e). As catfishes present a very developed sense of taste, aerial chemoreception in clariids was previously hypothesized to function mainly through the barbels [24], a gustatory organ. Furthermore, it was proposed that the nasal barbels might have co-opted the expression of olfactory receptors to increase their sensory capabilities. However, we only detected robust expression of the OLF repertoire in the olfactory epithelium and not in the barbels of the eel catfish using RNA-seq data (Fig. 3f). Thus, contrary to previous hypothesis, the sensory role of these receptor families is majorly restricted to smell and not taste in clariids.

Next, we studied whether fishes with large OLF gene repertoires present an augmented information-processing capacity in their olfactory bulbs. While frequently used as a proxy for smell functional abilities [6, 27], comparison of volume ratios in distantly related lineages is problematic as similarly sized brains can differ in the number and distribution of neurons [28]. Therefore, we dissected brain parts and estimated their cell number for twenty-four ray-finned fish species using the isotropic fractionator (see the “Methods” section). With this approach, we detected a positive correlation between the amount of receptor genes and the number of OB cells relative to other brain cells (PGLS, *p* = 0.0076, Fig. 3g). Importantly, freshwater amphibious nocturnal (FAN) fishes presented a higher fraction of OB cells compared to the rest of species, after correcting for phylogeny and brain size (*p* = 0.0004, Fig. 3h). Hence, our results link interspecific genetic variability with a phenotypic trait in the central nervous system and provide further evidence for enhanced olfactory abilities in amphibious lineages with expanded OLF gene repertoires.

### Alternative receptor expansions in amphibious fishes and tetrapods

**Fig. 4:**
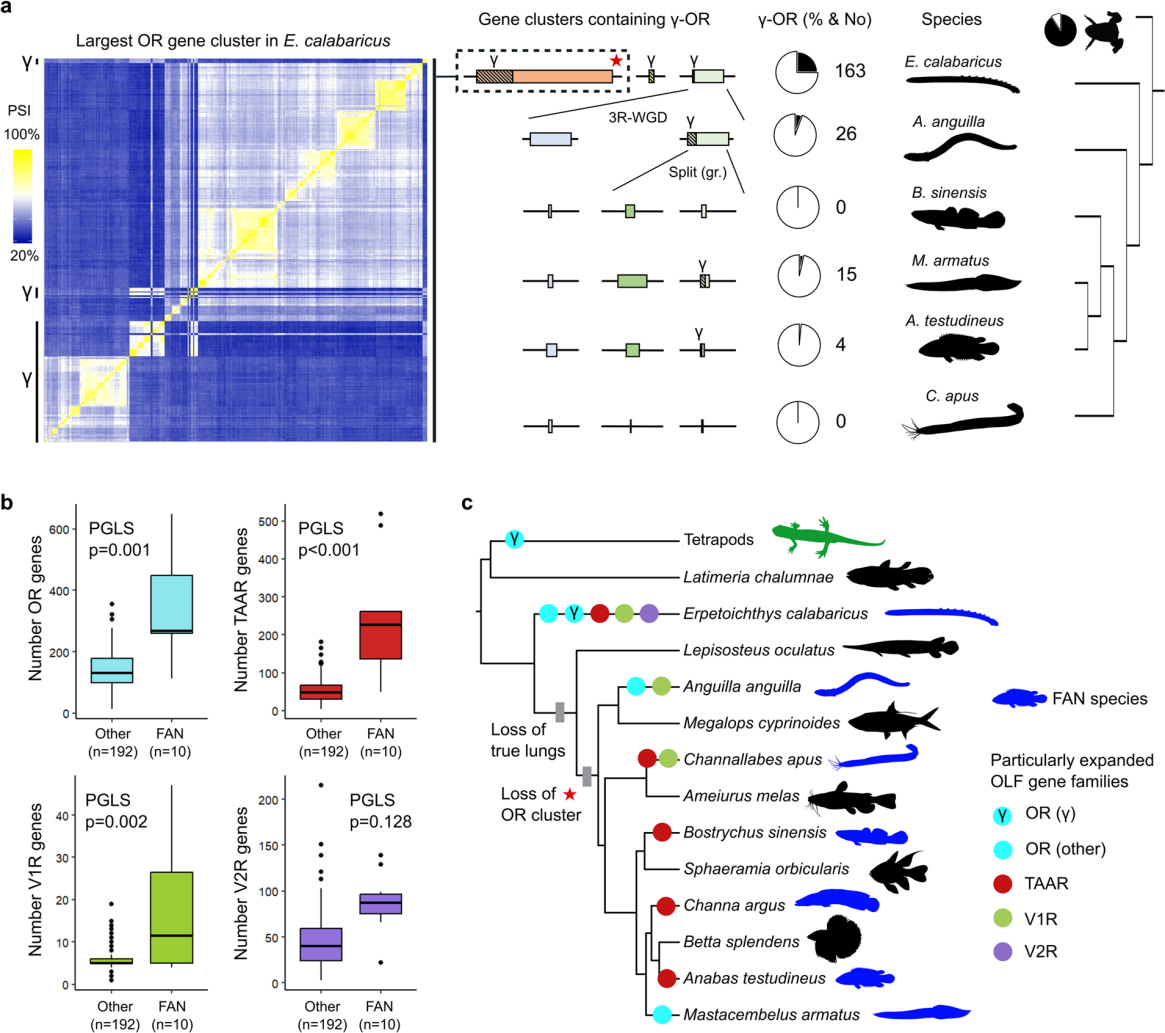
γ-OR genes scarcity in amphibious teleosts after gene cluster loss. **a** Evolutionary comparison of homologous gene clusters containing γ-OR genes in a subset of FAN species (one per taxonomic order), showing the highest number and proportion of this receptor subtype in the bichirs. Analysed teleost species present reduced or absent γ-OR repertoires, restricted to a unique gene cluster. Left, pairwise sequence identity (PSI) at the protein level between receptors from the largest gene OR cluster in the ropefish (*E. calabaricus*). Regions containing γ-OR genes are marked. Middle, boxes represent the relative size of gene clusters homologous to those containing γ-ORs in the bichirs. The proportion of γ-ORs (hatched) is also at scale. The third round of whole genome duplication (3R-WGD) at the base of the teleost clade and a genomic rearrangement (gr.) in clupeocephalans are indicated. The proportion of γ-OR in pie charts is calculated over the complete OR repertoire in each species. Dominant γ-OR proportion in an amphibian outgroup is also shown (*Xenopus tropicalis*). *E. calabaricus* and *Xenopus* silhouettes were downloaded from http://phylopic.org/. **b** Boxplots showing gene numbers for each of the OLF receptor families separately. Taking into account phylogeny (PGLS), significant differences are detected between FAN species (*n* = 10, Additional file 2: Table S8) versus the rest (*n* = 192) in three out of four OLF gene families (OR, TAAR and V1R). **c** Diagram depicting those OLF gene families particularly expanded in different FAN lineages (according to outlier species in Additional file 1: Fig. S2a and Fig. 3e). One representative organism for each taxonomic family that includes FAN species is shown. Gray boxes mark relevant events with potential influence in the proposed evolutionary scenario. A red star is used to reference the largest OR gene cluster found in the bichirs. Tetratpoda, *L. chalumnae*, *L. oculatus*, *M. cyprinoides*, *A. melas*, *S. orbicularis* and *B. splendens* silhouettes were downloaded from http://phylopic.org/

Last, we investigated whether gene expansions in some nocturnal amphibious fishes occurred in the same subtype of receptors most abundant in tetrapods, called γ-OR genes [11]. While they are scarce or even absent in amphibious teleost species, we observed a substantial number of γ-OR in the bichirs (Fig. 4a and Additional file 1: Fig. S4), in agreement with previous analysis [14]. Interestingly, we found the majority of γ receptors in this lineage located in a gene cluster that was lost before the teleost radiation, but still present in other ray-finned fishes. Additionally, while we detected significantly larger OR, TAAR and V1R gene repertoires in FAN species as a group compared to all other actinopterygians (PGLS, *p* < 0.01, Fig. 4b), phylogenetically separated FAN lineages present differences in the identity of the receptor families more affected by expansions (Fig. 4c), maybe linked to the evolution of greater sensitivity to distinct chemical compounds. In summary, increments in the number of chemoreceptors happened in different subtypes in most amphibious fishes compared to tetrapods, perhaps related to an ancient gene cluster loss in teleost ancestors without the sensory need to detect volatile odorants.

## Discussion

In this study, we identified independent expansions of OLF genes coding for chemosensory receptors in several lineages of ray-finned fishes. We also detected high global transcriptomic abundance in the case of expanded OLF gene clusters despite occasional regulatory biases enhancing the expression of individual genes from small clusters. Therefore, locus-restricted gene duplications might present an initial selective advantage by increasing the presence of certain receptor types in the olfactory epithelium. Moreover, we observed that duplicated copies tend to diversify their amino acid composition, as very few receptors present identical sequences in the studied fishes. This divergence might eventually lead to differences in their ligand affinity, activation threshold or even molecule recognition. If this functional disparity is indeed a frequent fate for duplicated receptors, species with OLF gene expansions would require an enhanced capacity in the brain to turn a more complex chemosensory input into profitable olfactory information. In this line, we found a significant correlation between the number of OLF genes and the relative amount of OB cells in the brain of twenty-four species with differently sized repertoires. While future detailed inspection of olfactory glomeruli will help to unveil the structural changes affecting enlarged OBs [29], we consider this finding to support the functional relevance of OLF gene expansions. Moreover, co-evolution between the number of lamellae in the olfactory rosette and the size of the OLF repertoire has been also reported in actinopterygians [14], suggesting additional morphological connections.

We also explored potential associations of several ecological and behavioural traits with olfaction-related changes across ray-finned fishes. We found that nocturnal amphibious lineages have convergently expanded their olfactory sensory system in terms of molecular receptors and OB processing capacity. This finding might be functionally related to the case of amphibians, which present distinct olfactory affinities in their aquatic and terrestrial forms and exceptionally extended OLF gene repertoires [30]. On the other hand, cetaceans and sea snakes have severely reduced their number of OR genes compared to land species [31], showing a consistent evolutionary response to the physical medium in vertebrates. Therefore, we suggest that these increments in the odour processing capacity of nocturnal amphibious fishes are potentially related to terrestrial orientation, especially in relatively large excursions during the search of new water bodies or prey. We further strengthened this evolutionary scenario with on purpose genomic and transcriptomic sequencing of a clariid species, an amphibious fish clade with aerial chemosensory abilities described in behavioural studies [24]. Nonetheless, we also identified relatively large gene repertoires in a reduced number of species from exclusively aquatic environments, indicating that additional ecological factors leading to olfactory variability in fishes remain to be discovered.

The exceptionally vast OLF gene repertoire found in the bichirs, the only ray-finned fishes retaining lung structures truly homologous to those of sarcopterygians [32], might reflect a deep history of aerial chemosensory abilities in this lineage. Interestingly, this clade presents a substantial number of genes belonging to the γ-OR subtype, the most abundant in land vertebrates. This group of chemoreceptors was considered to exclusively detect airborne odorants based on their virtual absence in the first studied fish genomes [11]. However, we hypothesize that the prevailing scarcity of this receptor subtype in most amphibious fishes might be related to the loss of an ancient γ-OR-containing gene cluster in primitive teleosts with limited olfactory abilities, rather than to putative functional futility in all types of aquatic habitats. In fact, this OR subtype is markedly present in non-teleost fishes such as the spotted gar or the coelacanth [33], suggesting an ancestral function in water environments [34]. Relatedly, it is increasingly recognised that many aquatic animals are able to detect molecular compounds that are both volatile and water-soluble [34, 35]. Moreover, some TAAR, V1R and (non-γ) OR receptors are reported to bind this type of chemical ligands, such as certain amines, small ketones and carboxylic acids [36–38]. Thus, we speculate that some of the chemosensory expansions found in amphibious fishes occurred in receptor genes already able to bind volatile molecules present in water. Finally, given that extant sarcopterygian fishes show modest OLF gene repertoires [39], an analogous magnification of the olfactory system might have happened among the first steps of the water-to-land transition in fish-like tetrapod ancestors able to move between water bodies. Therefore, we propose that chemosensory changes could precede many key morphological adaptations in one of the most crucial events during vertebrate evolution.

## Conclusions

The central finding of the present study is that nocturnal amphibious fishes have convergently expanded their olfactory system both genomically and in terms of brain processing capacity. As aquatic and terrestrial environments generally differ in their chemosensory cues, we suggest that such increments in the smell abilities of these fishes are likely related to the evolution of enhanced olfaction towards land exploration. Moreover, given that amphibious fishes can help unveil the first steps during the water-to-land transition of early tetrapods, we also consider our work relevant to understand the big picture of sensory evolution in vertebrates.

## Methods

### OLF repertoire annotation and analysis of gene clusters

We identified olfactory genes in 201 ray-finned fishes using publicly available high-quality genome assemblies (Additional file 2: Table S1). First, available reference protein sequences of OR, TAAR, V1R and V2R receptors from several species belonging to separate actinopterygian clades (spotted gar, zebrafish, stickleback, fugu, tongue sole, European seabass, Nile tilapia, mandarin fish, salmon and medaka) [8–11] were blasted against target genome assemblies (tblastn, *e*-value < 1e − 35). Only the best hits were recovered in case of overlapping queries. We built preliminary gene models grouping high scoring pairs belonging to different parts of the same queries to account for putative introns when necessary (maximum intron size of 10 Kb for OR, TAAR and V1R and 30 Kb for V2R). We extracted the genomic sequence spanning the gene models with an extension of three kilobases on each side. These sequences were processed with Exonerate (EMBL-EBI) using the original reference to generate optimized gene models (minimum identity of 35%). Next, we obtained the coding sequence of these new models with TransDecoder (https://github.com/TransDecoder/TransDecoder) and kept those equal or larger than 275 amino acids for OR, TAAR, V1R and 700 amino acids for V2R. The obtained sequences were blasted against a database containing OR, TAAR, V1R or V2R protein sequences and other G-protein coupled receptor families (HODER database) to filter out non-OLF receptors (blastp, *e*-value < 1e − 35). We repeated the whole process in each species using the new obtained gene models as references to further recover divergent unannotated OLF genes (Additional file 3: Data S1).

To avoid wrong estimates in our analysis, we restricted the dataset to publicly available high-quality genome assemblies. We only collected those assemblies that surpassed two a priori and one a posteriori filters: (i) The N50 metric divided by the total length of the genome was at least 10 Mb/Gb. (ii) The minimum required BUSCO.v3 completeness (actinopterygii10 database) was 90% for all species except for Polypteriformes, as a minimum of 85% was tolerated for the outgroup lineage within our dataset. (iii) To account for excessive genomic fragmentation, annotated OR genes needed to be distributed in less than twenty scaffolds or chromosomes, while in species with lineage-specific whole genome duplications up to thirty scaffolds were allowed.

To validate our annotation pipeline, we compared the number of OLF genes reported in Policarpo et al. [14] and this study (Additional file 1: Fig S1b-e). In particular, we contrasted the size of the OLF repertoires in a coincident subset of 112 species, obtaining a high correlation coefficient (Spearman's rho = 0.908). However, individual discrepancies were detected in some fishes when the studied genome assemblies were not the same, especially in cases with accentuated sequence fragmentation. In fact, a re-analysis using the same genome assemblies further increased the correlation (Spearman’s rho = 0.970), confirming the influence of sequence continuity in the estimation of OLF gene numbers. Still, we observed a few cases with notable differences, such as in *Labeo rohita* and *Gasterosteus aculeatus*, even when the same assemblies were analysed. In both species, we found the disparity in the reported size of the OLF repertoire mainly caused by the collapse of identical receptors performed in Policarpo et al. [14] (Additional file 1: Fig S1d). However, the two cases represent distinct situations. While the high number of identical receptors from *L. rohita* are probably consequence of bad haplotype resolution due to a low-quality genome assembly, the copies detected in *G. aculeatus* might correspond to real recent duplicates present in two distinct high-quality assemblies. Finally, we realised one last methodological difference putatively accounting for subtle differences in both studies: only monoexonic OR genes were annotated in Policarpo et al. [14] according to their procedure, excluding a small but existing fraction of intron-containing ORs in ray-finned fish species (Additional file 1: Fig. S1e).

Gene clusters were defined as genomic loci containing grouped receptor genes with a maximum intergenic distance between individual members not greater than a third of the whole cluster length measured in base pairs. Homologies among gene clusters in the studied species were inferred based on syntenic genes flanking the OLF genes (Additional file 2: Table S2). To unveil the cluster duplication dynamics, protein-level identity values from pairwise sequence alignments of analysed receptor genes were calculated with Clustal omega (EMBL-EBI). CD-HIT (https://github.com/weizhongli/cdhit) was used for grouping OLF genes with distinct levels of sequence identity (Additional file 1: Fig. S2c).

### Fish samples

Some of the studied species were obtained through the aquarium trade (Aquarium Glaser GmbH, Rodgau, Germany): *Anabas testudineus*, *Pygocentrus nattereri*, *Electrophorus electricus*, *Erpetoichthys calabaricus*, *Polypterus senegalus*, *Mastacembelus armatus*, *Xenentodon cancila*, *Thalassophryne amazonica*, *Gambusia affinis*, *Xiphophorus helleri* and *Channallabes apus*. Other fish were sampled from wild, semi-wild or captive facilities in the Czech Republic: *Acipenser ruthenus*, *Anguilla anguilla*, *Leuciscus idus*, *Rutilus rutilus*, *Gobio gobio*, *Esox lucius*, *Perca fluviatilis*, *Sander lucioperca*, *Oncorhynchus mykiss*, *Salmo trutta* and *Thymallus thymallus*. Last, a few marine species were obtained from fishing catches in Genoa (Italy): *Gadus morhua*, *Sparus aurata* and *Dicentrarchus labrax.* Two or three adult individuals from each species were processed for transcriptomic and/or brain analysis based on availability.

### Transcriptomic analyses

We dissected the olfactory organ from two individuals of eight species with diverse OLF gene repertoire sizes: *Anguilla anguilla* (669), *Pygocentrus nattereri* (530), *Electrophorus electricus* (332), *Thymallus thymallus* (85), *Salmo trutta* (183), *Thalassophryne amazonica* (141), *Anabas testudineus* (615) and *Mastacembelus armatus* (608). Total mRNA was extracted with RNeasy Mini Kit (Qiagen). RNA sequencing was performed by Novogene to obtain 30–55 million reads (paired end, 150 × 2 base pairs, Illumina platform) per sample (Additional file 2: Table S3). Reads were filtered with Fastp software [40]. We estimated the expression of annotated receptors by balancing the number of uniquely and ambiguously mapped reads using Salmon [41]. As each sensory neuron expresses only one receptor, and mRNA levels are known to correlate with the number of neurons expressing a given receptor [2, 42], we calculated the relative abundance of every transcript among all OLF receptors for interspecific comparison. This approach also reduced potential bias due to contamination of surrounding non-olfactory tissues compared to absolute measurements. Mean values obtained from biological replicates for each gene were used. A particular V2R gene located outside of the main gene cluster of this family and lacking receptor activity is known to be co-expressed with other V2R genes in previously studied vertebrates [43]. Accordingly, we identified homolog singletons of this gene with very high expression in our studied fish species. Hence, we decided to eliminate it from the expression analysis to avoid overestimation in V2R abundance. In the case of *Channallabes apus* analysis, read counts per million were used for direct comparison of the expression level of OLF genes among tissues.

### Time-calibrated phylogenetic tree

We downloaded the protein sequences of 640 exons from zebrafish (Ensembl), selected by a previous study as optimal for phylogenomic analysis in teleost fishes [44]. Next, we blasted these exons against all the genome assemblies in our dataset (blastn, *e*-value < 1e − 50). We retrieved the sequences corresponding to the best hits and blasted them back to the zebrafish genome (GRCz10). Those exons with reciprocal best hit were considered homologous and used in the subsequent steps. Homologous exons were aligned with MAFFT [45] (using the accurate option L-INS-i) and the two first and last nucleotides were trimmed from each alignment to avoid potential gaps caused by incomplete sequence retrieval near the splice sites. The nucleotide sequences of the exon alignments were concatenated and a maximum-likelihood phylogenetic analysis with a topological constrain for non-teleost species following current molecular phylogenies [46] was conducted using IQTREE2 [47] (-B 10000 -abayes -alrt 1000) with the substitution model (GTR + F + R10) chosen according to Bayesian Information Criterion. To generate a time-calibrated phylogeny (Additional file 4: Time-calibrated phylogeny), the obtained tree was processed with BEASTv2.5 applying the UCLN relaxed clock model, independent GTR site models with γ-distributed rate variation and the birth–death tree prior with an age constraint on the root node set to 375 Mya and a SD of 20 My.

### Analysis of ecological features

We gathered information from public databases and scientific literature for several ecological parameters with a potential impact on the evolution of smell abilities (Additional file 2: Table S4). Information involving habitat zone (Pelagic, Benthic, Benthopelagic, Reef-associated), salinity (Marine, Freshwater, Brackish) and diadromous migrations was mainly collected from FishBase using “rfishbase” R package. Amphibious behaviour was determined following available studies [19, 20]. Data regarding nocturnal, arrhythmic or diurnal activity was obtained from the literature for approximately 80% of species in our dataset (Additional file 2: Table S5). Species presenting a nocturnal/crepuscular pattern were classified as nocturnal, while those having a diurnal/crepuscular pattern were considered diurnal in our analysis. To assess the effect of the studied ecological parameters on the size of the OLF gene repertoires, we used Bayesian phylogenetic multilevel models using the “brms” package [48]. The response variable was log10-transformed number of OLF genes and the explanatory variables were the different ecological categories (Additional file 2: Table S6). We performed robust linear regression using the student family, specifying the following weakly informative priors: prior(normal(0, 1), “b”), prior(normal(2, 0.5), “Intercept”), prior(student_t(3, 0, 1), “sd”), prior(student_t(3, 0, 1), “sigma”), prior(gamma(3, 0.1), “nu”). Prior predictive checks confirmed that these priors set reasonable expectations for the model coefficients. To check for prior sensitivity, we also ran the model with default priors and the model inference was not changed. We ran 4 MCMC chains for 20,000 iterations with a warm-up period of 10,000 and thinning every 10 steps, resulting in 4000 post-warmup draws. Proper chain mixing was checked by visually inspecting the trace plots. Additionally, Rhat for all parameters was < 1.003 and the bulk and tail effective sample sizes for all parameters were at least 500 and typically over 80% of total post-warmup draws, indicative of model convergence. Bayesian *R*^2^ was calculated using the bayes_R2 function in the brms package. The phylogenetic signal was estimated using the hypothesis function in the brms package, following the brms vignette (https://cran.r-project.org/web/packages/brms/vignettes/brms_phylogenetics.hhtm). Interestingly, analysing the OLF gene families separately produced comparable trends to those obtained with the whole repertoire for most parameters (Additional file 1: Fig. S3), revealing overall similar dynamics in their response to ecological factors.

### *Channallabes apus* genome sequencing

Genomic DNA (gDNA) of *Channallabes apus* was extracted with the circulomics Nanobind Tissue Big DNA kit according to the manufacturer’s instructions. In brief, liver tissue was minced to small slices on a clean and cold surface and finally homogenized with the TissueRuptor II device (Qiagen) making use of its maximal settings. After complete tissue lysis, remaining cell debris was removed, and the gDNA was bound to circulomics Nanobind discs in the presence of Isopropanol. High molecular weight (HMW) gDNA was eluted from the nanobind discs in elution buffer (EB). The integrity of the HMW gDNA was determined by pulse field gel electrophoresis using the Femto Pulse device (Agilent Technologies), showing a clear peak 127 kb in length. All pipetting steps of ultra-long and long gDNA were done carefully with wide-bore pipette tips.

The HMW gDNA was managed as recommended by Pacific Biosciences according to the guidelines using the SMRTbell Express Template Prep Kit 2.0 (PN 101–853-100, version 03). In summary, gDNA was sheared twice with the MegaRuptorTM device (Diagenode) applying the 25 and 20 kb shearing options. 5 ug sheared gDNA went into library preparation. Size selection was performed for fragments larger than 5.5 kb with the BluePippinTM device, resulting in a library of 11.8 kb in size (Fragment Analyzer, Agilent Technologies). The size selected library ran on one Sequel II SMRT cells for 30 h.

Chromatin conformation capturing of *Channallabes apus* chromatin was performed with the ARIMA Hi-C + Kit (Material Nr. A410110). In brief, circa 50 mg of flash-frozen powdered tissue were chemically crosslinked in enriched nuclei. The crosslinked genomic DNA was digested with the restriction enzyme cocktail consisting of four restriction enzymes. The 5′-overhangs were filled in and labelled with biotin. Spatially proximal digested DNA ends were ligated. The ligated biotin-containing fragments were enriched and used for Illumina library preparation, with the Kapa Hyper Prep kit (ARIMA Document Part Number A160139 v00). The barcoded Hi-C library run on an S4 flow cell of a NovaSeq6000 with 2 × 150 cycles.

### *Channallabes apus* genome assembly

We created PacBio CCS reads (read quality > 0.99) from the *Channallabes apus* subreads.bam file using PacBio’s ccs command line tool (version 6.3.0). We obtained 28.36 Gb high-quality CCS reads (HiFi reads) with a N50 of 11.58 Kb. To further increase the read quality and coverage, we applied the tool DeepConsensus (v0.2, on PacBio reads within 98.0–99.5% read accuracy) [49] and gained an overall yield of 30.02 Gb (N50: 11.63 Kb). PacBio reads containing the PacBio adapter sequence were filtered out by applying a blast [50] search providing the adapter sequence and the following arguments “reward 1 -penalty -5 -gapopen 3 -gapextend 3 -dust no -soft_masking false -evalue 700 -searchsp 1750000000000 -outfmt 7”. Initial contigs were generated using HiFiasm (v0.16.1-r375) [51] with parameters –primary -l0 and alternative haplotigs were purged using purge-dups (v1.2.3) [52]. To create the set of alternative contigs, purge-dups was also run on the alt assembly from hifiasm combined with the purged output of running purge-dups on the primary contigs. Initial scaffolding of the primary assembly was performed by mapping Hi-C reads to the primary contigs using bwa-mem (v0.7.17-r1198-dirty) and mappings were filtered following the VGP arima mapping pipeline (https://github.com/VGP/vgp-assembly/tree/master/pipeline/salsa). The final bed file was given to yahs [53] for scaffolding.

In order to correct any false or missed joins made in the automated scaffolding process, the HiC viewer HiGlass (v2.1.11) was used to visually inspect and curate the assembly into chromosomes. This followed an iterative process of re-ordering and re-orienting the assembly until no further corrections or incorporations of unscaffolded sequence into chromosomes could be made to the assembly. Finally, the primary and alternative assemblies were polished by mapping the HiFi reads to the assemblies using pbmm2 (v1.3.0) [https://github.com/PacificBiosciences/pbmm2.git] with arguments –preset CCS -N 1 and variants called using deepvariant (v0.2.0) with –model_type = PACBIO. Finally, errors were corrected in the assembly by filtering the vcf file given by deepvariant (v0.2.0) [54] with bcftools view (v1.12) [55] with arguments -i 'FILTER = \"PASS\" && GT = \"1/1\"' and a consensus called with bcftools consensus. Finally, we obtained a primary genome assembly of approximately 1.16 Gb in size, 102 scaffolds and a N50 of 42.72 Mb.

### Brain dissection, cell count and PGLS analysis

Fish were anesthetized in a tricaine methanesulfonate (MS-222) solution (500 mg/l) and their body mass and length were measured. After additional intramuscular injection of a ketamine/xylazine mixture (3:1), they were perfused transcardially with wormed phosphate buffered saline containing 0.1% heparin followed by cold 4% paraformaldehyde solution. The dorsal part of the skull was largely removed and the head with exposed brain was fixed in the 4% paraformaldehyde for about 30–60 min. The brain was then dissected and weighed using a Mettler Toledo MX5 microbalance (Mettler Toledo, Columbus, Ohio). Brains were postfixed for additional 2–3 days, transferred to an antifreeze solution (30% glycerol, 30% ethylene glycol, 40% phosphate buffer) and kept frozen at 20 °C until processing.

Olfactory bulbs were separated from the rest of the brain. The two parts were weighed and the total number of their constituent cells was determined following the procedure of isotropic fractionator [56]. Briefly, each brain division was homogenized in a dissociation solution (40 mM sodium citrate solution with 1% Triton X-1000) using glass tissue grinders (0.5 ml or 1 ml, Ningbo Ja-Hely Technology Co., Ltd., China). When turned into an isotropic suspension of free cell nuclei, homogenates were stained with the fluorescent DNA marker 4′,6-Diamidino-2-phenylindole dihydrochloride (DAPI) (Sigma-Aldrich); its volume was determined using an Eppendorf Xplorer 5–1000 μL electronic pipette (Eppendorf, Hamburg, Germany) and kept homogenous by agitation. The total number of nuclei in suspension, and therefore the total number of cells in original tissue, was estimated by determining the number of nuclei in 10 μl samples drawn from the homogenate (Additional file 2: Table S7a-b). At least six aliquots were sampled and counted using a Neubauer improved counting chamber (BDH, Dagenham, Essex, UK) at the AxioImager.A2 microscope (Carl Zeiss AG, Jena, Germany) equipped with epifluorescence and appropriate filter settings; additional aliquots were assessed when needed to reach the coefficient of variation among counts ≤ 0.1.

To assess the relationship between the number of olfactory genes and the number of olfactory bulb cells, we performed phylogenetic least squares regression using the gls function in the R package nlme [57] (R package version 3.1–159, https://CRAN.R-project.org/package=nlme). The response variable was the number of cells in the olfactory bulbs and the explanatory variables were the number of cells in the rest of the brain (to control for overall brain size) and the number of OLF genes. All variables were log10-transformed prior to analysis. To visualize the relationship, the residuals from regression of olfactory bulb cells on the rest of brain cells were plotted against the number of OLF genes.

## Supplementary Information

**Additional file 1: Fig. S1.** OLF gene repertoire contractions in ray-finned fishes and annotation comparison with a previous study. **Fig. S2.** OLF gene families present disparate dynamics in species with expanded repertoires. **Fig. S3.** Ecological factors influencing the evolution of OR, TAAR, V1R and V2R gene repertoires in ray-finned fishes. **Fig. S4.** Phylogenetic analysis of OR subtypes in ray-finned fishes with large OLF gene repertoires.

**Additional file 2: Table S1.** Number of OLF genes identified in genome assemblies. **Table S2.** Gene cluster homologies in species with expanded OLF repertoires. **Table S3.** Information of transcriptomic samples. **Table S4.** Ecological traits in analysed ray-finned fish species. **Table S5.** Classification of diurnal and nocturnal activity in fishes. **Table S6.** Statistical comparison of different ecological, life-history and behavioural traits. **Table S7.** Number of cells in the olfactory bulb and brain of twenty-four fish species. **Table S8.** Complete list of FAN species.

**Additional file 3: Data S1.** Protein sequences and genomic coordinates of OLF genes. FASTA and General Feature Format (GFF) files containing the protein sequences and genomic coordinates for all identified OLF genes in this study.

**Additional file 4.** Time-calibrated phylogenetic tree of the studied ray-finned fish species used in the Bayesian phylogenetic multilevel models analysis.

## Data Availability

All data generated or analysed during this study are included in this published article, its supplementary information files and publicly available repositories. The eel catfish genome assembly and associated raw data can be found with the following accession codes: Primary assembly: BioProject accession PRJNA834615, Genome accession JAMASW000000000. Alternate assembly: BioProject accession PRJNA834614, Genome accession JAMASX000000000. Biosample: SAMN28052917. PacBio HiFi data: SRA accession SRR19049190. Hi-C data: SRA accession SRR19049189. All fastq files of the RNA-seq samples generated for this project are available at SRA within BioProject PRJNA899054. Other data generated for this project can be found in Additional files 2 and 3.
