# Supplementary material for: Expanded olfactory system in ray-finned fishes capable of terrestrial exploration
Source: BMC Biol. 2023 Jul 31;21:163. doi: 10.1186/s12915-023-01661-8 (PMC10392011; doi:10.1186/s12915-023-01661-8)
Supplement: Supplementary file 1 — Additional file 1: Fig. S1. OLF gene repertoire contractions in ray-finned fishes and annotation comparison with a previous study. Fig. S2. OLF gene families present disparate dynamics in species with expanded repertoires. Fig. S3. Ecological factors influencing the evolution of OR, TAAR, V1R and V2R gene repertoires in ray-finned fishes. Fig. S4. Phylogenetic analysis of OR subtypes in ray-finned fishes with large OLF gene repertoires. [file 12915_2023_1661_MOESM1_ESM.pdf]

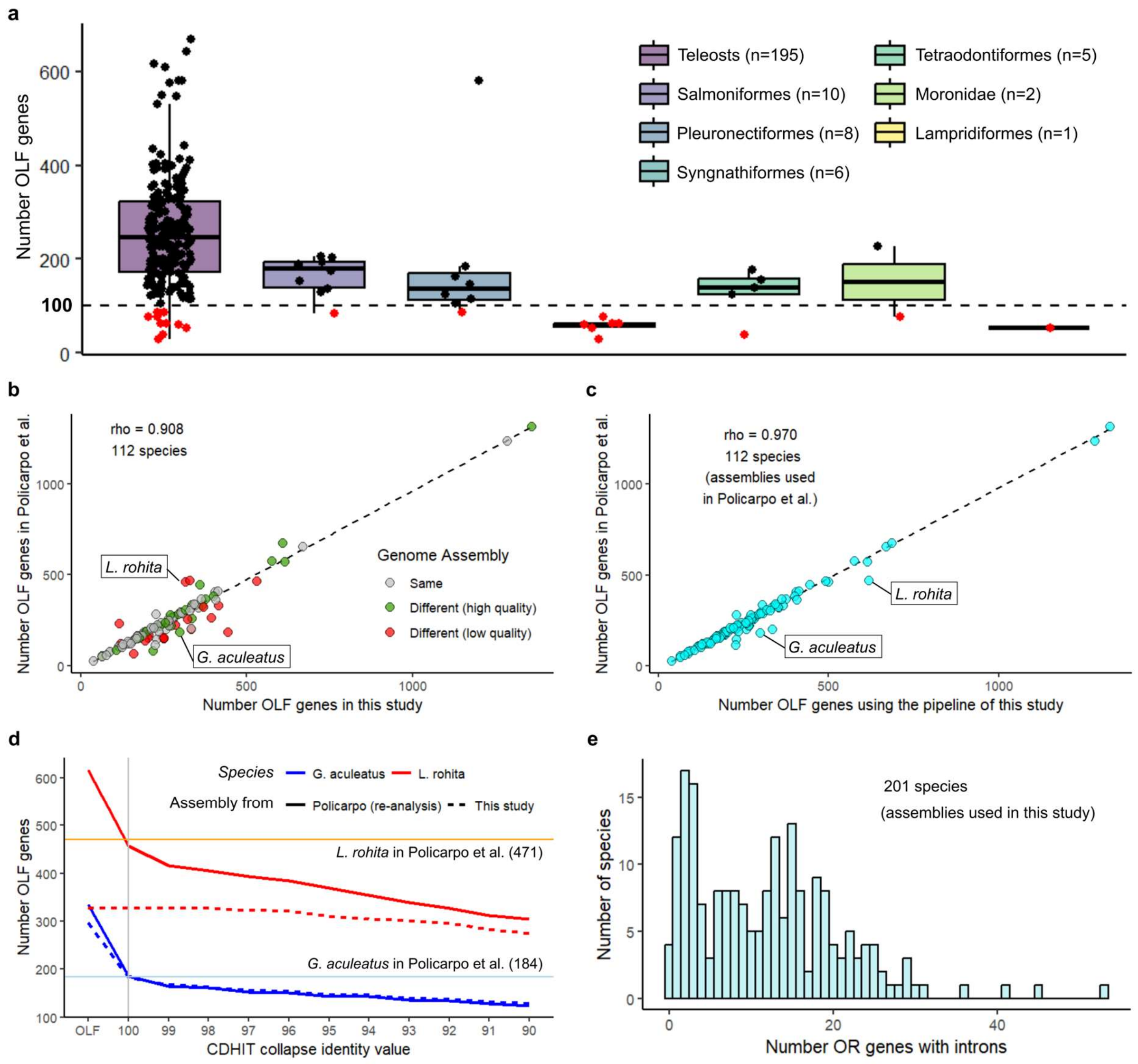

Fig. S1: OLF gene repertoire contractions in ray-finned fishes and annotation comparison with a previous study. (a) Boxplots showing the number of OLF genes in the clades containing species with less than 100 receptor genes (in red). All individual species from the characterized lineages are depicted by dots. The complete teleost dataset is also included to provide a wider phylogenetic context. **(b)** Comparison of the total number of OLF genes reported in Policarpo et al. 2022 and this work in 112 overlapping species. Grey colour is used when the same genome sequence assembly was analysed in both studies. Green dots represent species in which two different assemblies were analysed, both considered high-quality (such as *G. aculeatus*). Red colour is devoted for those species where the genome assemblies inspected in Policarpo et al. 2022 do not meet the minimum quality requirements used in this study (such as *L. rohita*). **(c)** Additional comparison between the number of OLF genes reported in Policarpo et al. 2022 and the number of detected receptors when analysing the same genome sequence assemblies using our annotation pipeline. **(d)** Number of OLF genes detected in two distinct genome assemblies (from Policarpo et al. 2022 and this study) of *G. aculeatus* and *L. rohita* using our annotation pipeline. The number of OLF genes after successive sequence identity collapses employing CDHIT is included from 100% to 90% identity percentage. The number of OLF genes reported by Policarpo et al. 2022 for those species is also shown with horizontal lines. **(e)** Histogram showing the proportion of species presenting a given number of intron-containing OR genes according to this study.

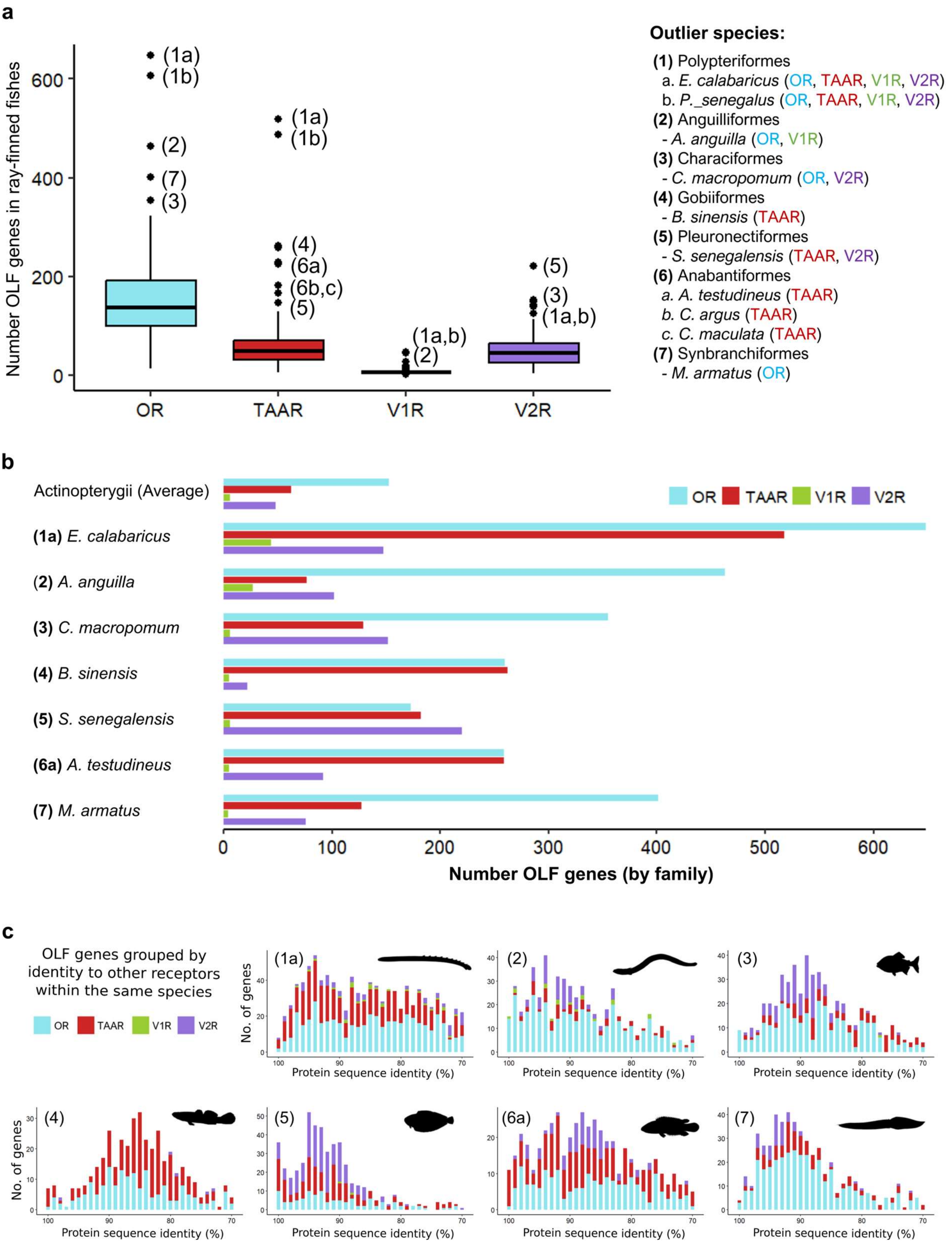

Fig. S2: OLF gene families present disparate dynamics in species with expanded repertoires. (a) Boxplots showing the distribution of the repertoire size for each of the OLF gene families (OR, TAAR, V1R and V2R) in the studied ray-finned fishes (n=201). In general, OR genes constitute the largest family, followed by TAAR and V2R genes with intermediate family sizes, being the V1R genes the smallest group. Right, a legend identifying the outlier species presenting particularly expanded repertoires in one or more OLF gene families. **(b)** Bar plot separately showing the number of annotated

receptors from OR, TAAR, V1R and V2R gene families in those species highlighted in Figure 1a. **(c)** Number of receptor genes with a given percentage of sequence similarity at the protein level relative to other genes for the species shown in the previous figure panel. Genes presenting identity values lower than 70% are excluded from the plots. While most species have a small number of identical receptors, differences in the duplication dynamics among OLF gene families are observed.

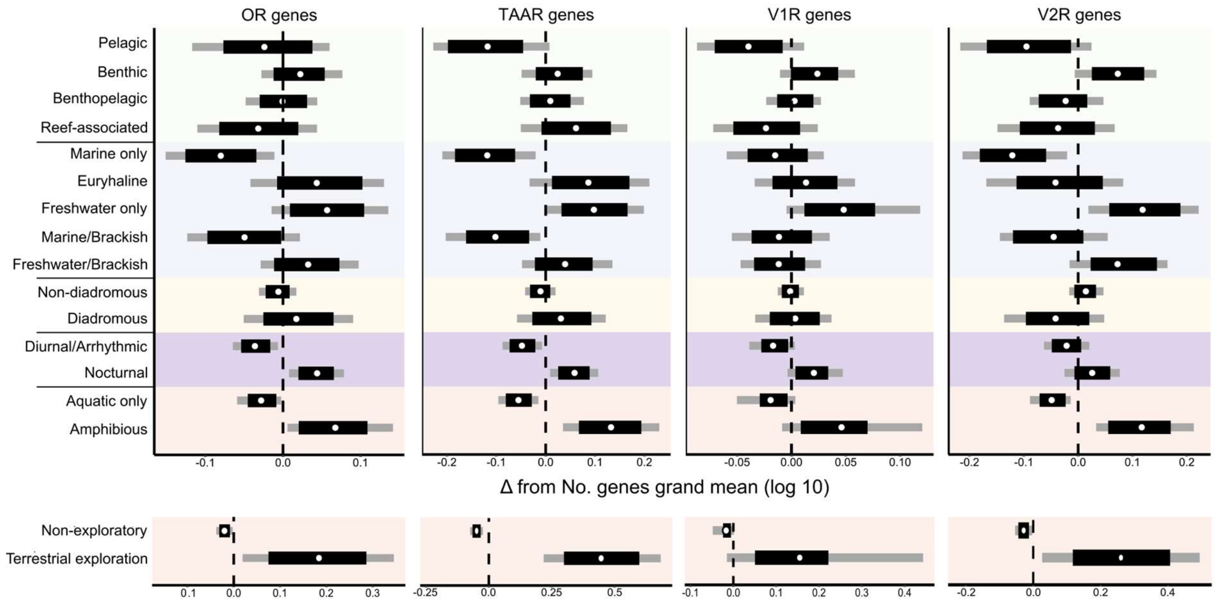

Fig. S3: Ecological factors influencing the evolution of OR, TAAR, V1R and V2R gene repertoires in ray-finned fishes. Complementary analysis (Figure 3a), showing the effect size distribution of several ecological traits on the number of OR, TAAR, V1R and V2R genes, separately. Highest density intervals (HDI) of 80% (black) and 95% (grey). Colours delimit related factors containing exclusive sets of species.

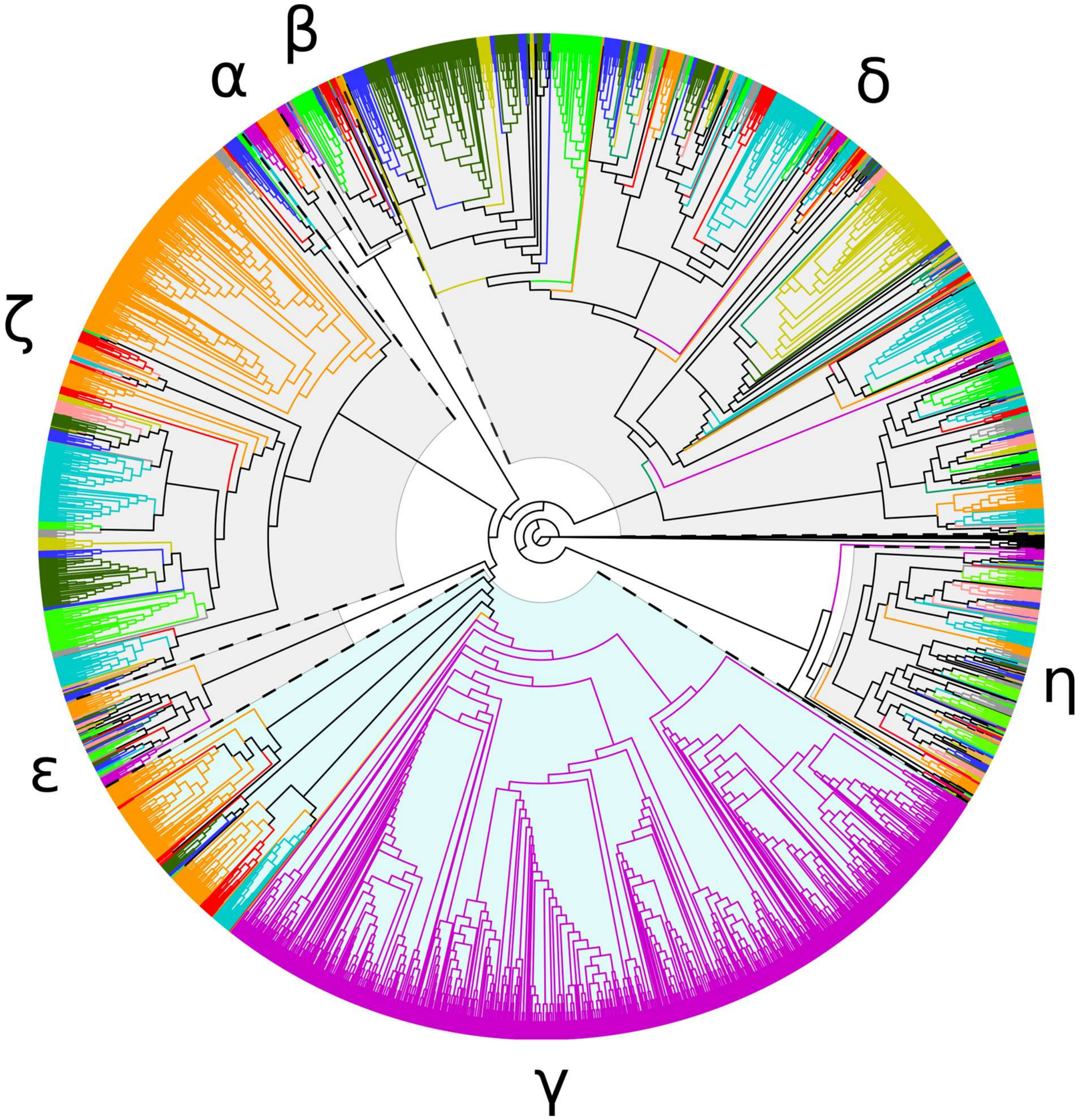

Fig. S4: **Phylogenetic analysis of OR genes subtypes in ray-finned fishes with large OLF repertoires.** Maximum-likelihood phylogenetic tree containing OR protein sequences from those species with more than 500 OLF genes highlighted in Fig. 1, plus spotted gar, zebrafish, and three tetrapods (*Gallus gallus*, *Anolis carolinensis* and *Xenopus tropicalis*) obtained from public sources. Length of branches is transformed, with tips aligned to help visualization. Distinct branch colours are used for genes from each species or lineage. Colour identities are as follow: tetrapod species in light purple, ropefish (*Erpetoichthys calabaricus*) in orange, spotted gar (*Lepisosteus oculatus*) in red, European eel (*Anguilla anguilla*) in aquamarine, tambaqui (*Colossoma macropomum*) in light green, zebrafish (*Danio rerio*) in grey, four-eyed sleeper (*Bostrychus sinensis*) in dark yellow, Senegalese sole (*Solea senegalensis*) in pink, climbing perch (*Anabas testudineus*) in dark blue and zig-zag eel (*Mastacembelus armatus*) in dark green. OR subtypes are indicated with their corresponding greek letter in the outer part: alpha (α), beta (β), gamma (γ), delta (δ), epsilon(ε), zêta (ζ), êta (η). γ-OR receptors are highlighted in light blue background. Sequences were aligned with MAFFT and IQTREE2 was used for phylogenetic reconstruction.
