# Supplementary figures and images for: Expanded olfactory system in ray-finned fishes capable of terrestrial exploration
Source: BMC Biol. 2023 Jul 31;21:163. doi: 10.1186/s12915-023-01661-8 (PMC10392011; doi:10.1186/s12915-023-01661-8)

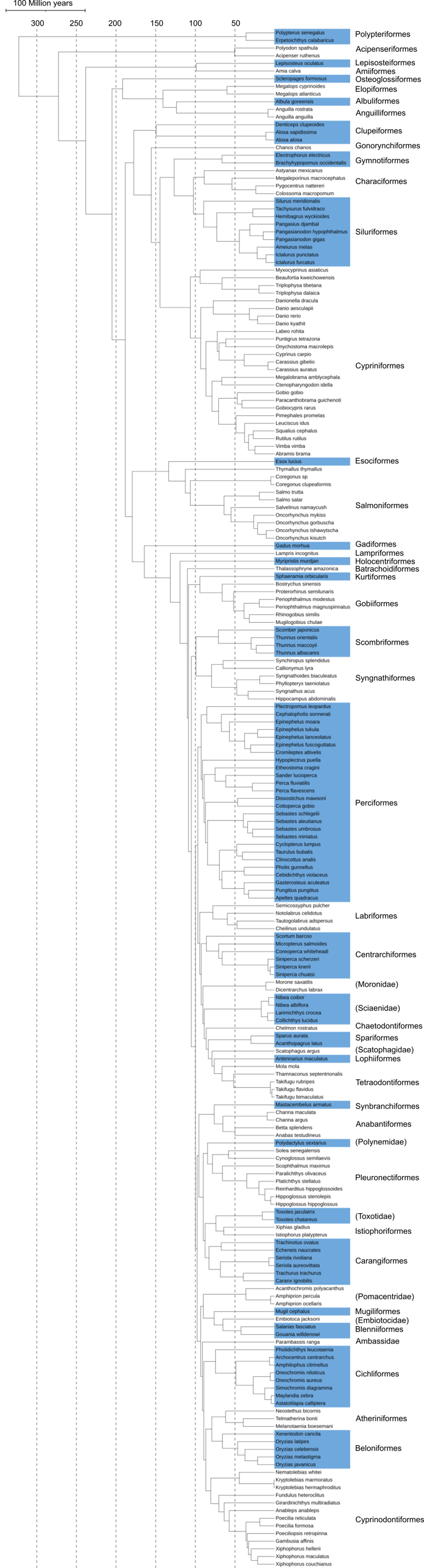

Supplement: Supplementary file 4 — Additional file 4. Time-calibrated phylogenetic tree of the studied ray-finned fish species used in the Bayesian phylogenetic multilevel models analysis. [file 12915_2023_1661_MOESM4_ESM.png]
